# Radiosensitizing effect of lapatinib in human epidermal growth factor receptor 2-positive breast cancer cells

**DOI:** 10.18632/oncotarget.12597

**Published:** 2016-10-12

**Authors:** Tosol Yu, Bong Jun Cho, Eun Jung Choi, Ji Min Park, Dan Hyo Kim, In Ah Kim

**Affiliations:** ^1^ Department of Radiation Oncology, Graduate School of Medicine, Seoul National University, Seoul, Republic of Korea; ^2^ Medical Science Research Institute, Seoul National University Bundang Hospital, Seongnam, Seoul, Republic of Korea; ^3^ Department of Radiation Oncology, Seoul National University College of Medicine, Seoul, Republic of Korea; ^4^ Cancer Research Institute, Seoul National University College of Medicine, Seoul, Republic of Korea; ^5^ Institute of Radiation Medicine, Seoul National University College of Medicine, Seoul, Republic of Korea

**Keywords:** breast cancer, human epidermal growth factor receptor 2, radiotherapy, lapatinib

## Abstract

Trastuzumab has been widely used for the treatment of human epidermal growth factor receptor 2 (HER2)-overexpressing breast cancer, however, it cannot easily cross the blood-brain barrier (BBB) and is known to increase the incidence of brain metastases. In contrast, lapatinib has a low molecular weight and can cross the BBB and it could be useful to treat brain metastases in patients with HER2-positive breast cancer.

To explore the impact of lapatinib on radiation response, we conducted an *in vitro* experiment using SKBR3 and BT474 breast carcinoma cells exhibiting HER2/neu amplification. Lapatinib down-regulated phosphorylated (p)-HER2, p-epidermal growth factor receptor, p-AKT, and p-extracellular signal-regulated kinase. Pretreatment of lapatinib increased the radiosensitivity of SKBR3 (sensitizer enhancement ratio [SER]: 1.21 at a surviving fraction of 0.5) and BT474 (SER: 1.26 at a surviving fraction of 0.5) cells and hindered the repair of DNA damage, as suggested by the prolongation of radiation-induced γH2AX foci and the down-regulation of phosphorylated DNA-dependent protein kinase, catalytic subunit (p-DNAPKcs). Increases in radiation-induced apoptosis and senescence were suggested to be the major modes of cell death induced by the combination of lapatinib and radiation. Furthermore, lapatinib did not radiosensitize a HER2- negative breast cancer cell line or normal human astrocytes.

These findings suggest that lapatinib can potentiate radiation-induced cell death in HER2-overexpressing breast cancer cells and may increase the efficacy of radiotherapy. A phase II clinical trial using lapatinib concurrently with whole-brain radiation therapy (WBRT) is currently being conducted.

## INTRODUCTION

The human epidermal growth factor receptor 2 (HER2) is overexpressed in about 20 to 30% of breast carcinomas and is associated with aggressive clinical course [1]. The introduction of trastuzumab, an anti-HER2 monoclonal antibody, has dramatically improved the survival of HER2-positive patients [2–5]. However, the incidence of brain metastasis is significantly increased in HER2-positive breast cancer after trastuzumab treatment. This may be due to the fact that trastuzumab enhances systemic control and prolongs survival, thus clinically disclosing brain metastasis [6]. Trastuzumab is not easily cross the blood-brain barrier (BBB) due to its large molecular weight (145,531 Dalton). Despite receiving trastuzumab-based therapy, approximately 30% of patients with HER2-positive metastatic breast cancer develop brain metastasis, and intracranial disease progression, rather than extracranial disease, is the main cause of death in those patients [7–10].

Lapatinib ditosylate is a reversible dual inhibitor of the intracellular tyrosine kinase domain of HER1 (also known as epidermal growth factor receptor, EGFR) and HER2 [11]. Lapatinib has a very low molecular weight (581 Da), and its theoretical ability to cross the blood-brain barrier makes it an ideal candidate for testing against brain metastases [12]. Preclinical evidence supports the activity of lapatinib against CNS disease. Lapatinib is the first HER2-targeting drug to be validated in a preclinical model for activity against brain metastasis from HER2-positive breast cancer [13].

There have been attempts to demonstrate the effect of lapatinib on brain metastasis in HER2-positive breast cancer patients, however, the response rate of lapatinib alone for brain metastasis was utmost 3–6% [14–20].

We hypothesized that the combination of radiotherapy (RT) and lapatinib could be an effective strategy for treating HER2-positive breast cancer. Although several *in vitro* studies have used lapatinib alone or in combination with other chemotherapeutic agents, there are few *in vitro* studies regarding the combined effect of lapatinib with RT [13, 21]. In this study, we investigated the radiosensitizing effect of lapatinib by using HER2-positive breast cancer cell lines and tried to identify the mechanism of interaction and the modes of cell death by lapatinib combined with radiation. We also assessed the effect of lapatinib on HER2-negative breast cancer cells and normal human astrocytes.

## RESULTS

### Lapatinib radiosensitized HER2-overexpressing breast cancer cells

**Figure 1:**
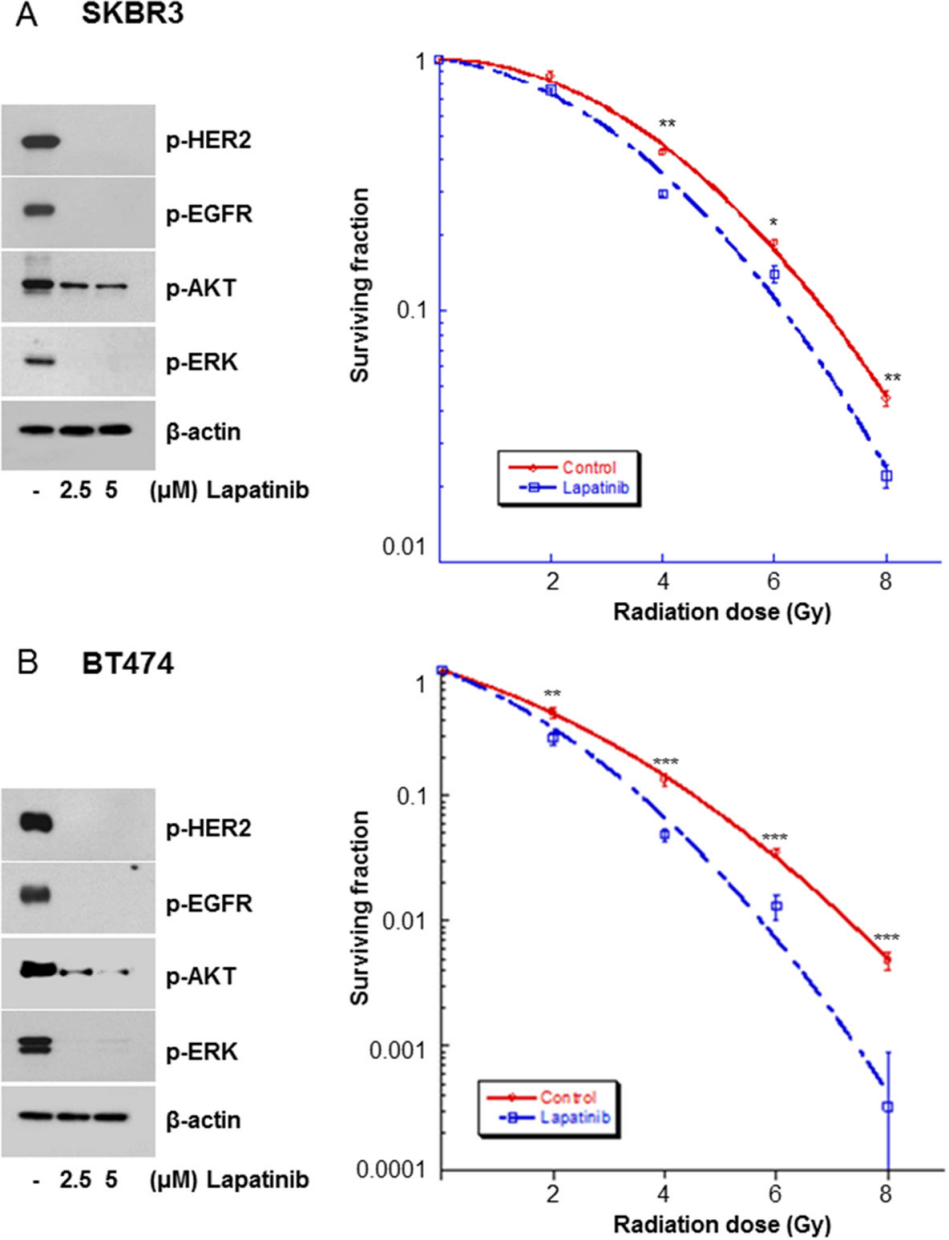
Lapatinib radiosensitized HER2-overexpressing breast cancer cells **A.** SKBR3 cells were treated with different concentrations of lapatinib, and the cell extracts were western blotted with the indicated antibodies. Lapatinib attenuated the expression of phosphorylated (p)-HER2, p-epidermal growth factor receptor (EGFR), p-AKT, and p-extracellular signal-regulated kinase (ERK) in SKBR3 cells. SKBR3 cells were also treated with either dimethyl sulfoxide (DMSO) or lapatinib (5 μM) and irradiated with 4-MV x-rays. The surviving fraction of lapatinib-treated cells was lower than that of the control group. **B.** BT474 cells were treated with different concentrations of lapatinib, and the cell extracts were western blotted with the indicated antibodies. Lapatinib attenuated the expression of p-HER2, p-EGFR, p-AKT, and p-ERK in BT474 cells. BT474 cells were then treated with either DMSO or lapatinib and irradiated with 4-MV x-rays. The surviving fraction of lapatinib-treated cells was lower than that of the control group. ^*^P≤0.05. ^**^P≤0.01. ^***^P≤0.001.

To explore the impact of lapatinib on radiation response, SKBR3 and BT474 breast carcinoma cells exhibiting HER2/neu amplification were treated with DMSO or 5 μM lapatinib. Lapatinib resulted in the down-regulation of p-HER2, p-EGFR, and p-ERK (Figure 1A and 1B). This was associated with an increase in the radiosensitivity of SKBR3 and BT474 cells, as shown by the clonogenic assays (Figure 1). In SKBR3 cells, the SERs of lapatinib at surviving fractions of 0.5 and 0.05 were 1.21 and 1.11, respectively. In BK474 cells, the SERs at surviving fractions of 0.5 and 0.05 were 1.258 and 1.279, respectively.

### Lapatinib hindered the repair of DNA damage

**Figure 2:**
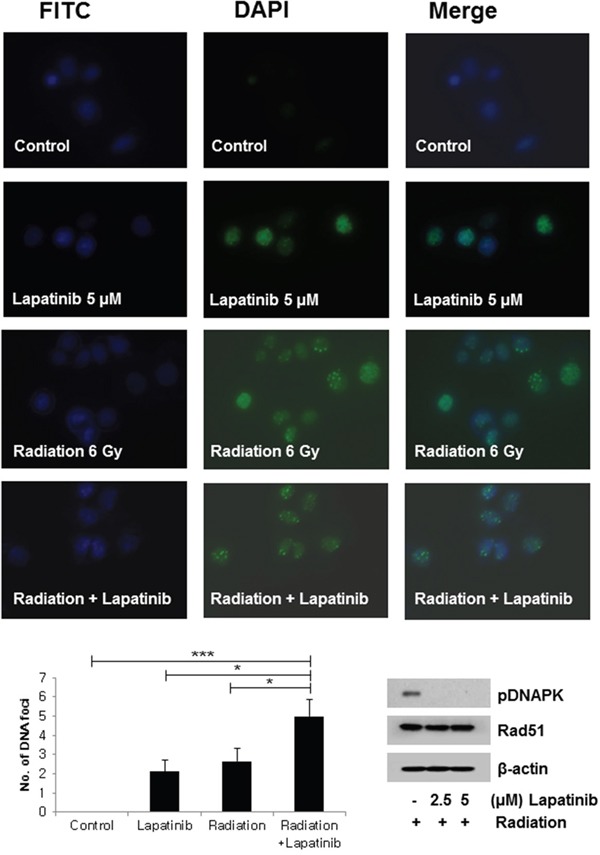
DNA damage repair: Lapatinib induced an increase in γH2AX foci SKBR3 cells were treated with DMSO, lapatinib (5 μM), radiation (6 Gy), or both lapatinib (5 μM) and radiation (6 Gy). The degree of DNA damage repair was determined by the number of formed γH2AX foci at 24 hours following treatment of lapatinib or radiation. Lapatinib increased the formation of γH2AX foci. Whole-cell extracts were western blotted with p-DNAPK, Rad51, and β-actin. The expression of p-DNA-PKcs was down-regulated after lapatinib treatment. ^*^P≤0.05. ^***^P≤0.001.

Pretreatment of lapatinib markedly increased the number of radiation-induced γH2AX foci, indicating a delayed repair of DNA damage compared to control (Figure 2). Lapatinib led down-regulation of p-DNAPK_cs_, which is mainly involved in NHEJ repair. However, Rad51, which represents homologous recombination repair, was not affected by lapatinib.

### Modes of cell death

**Figure 3:**
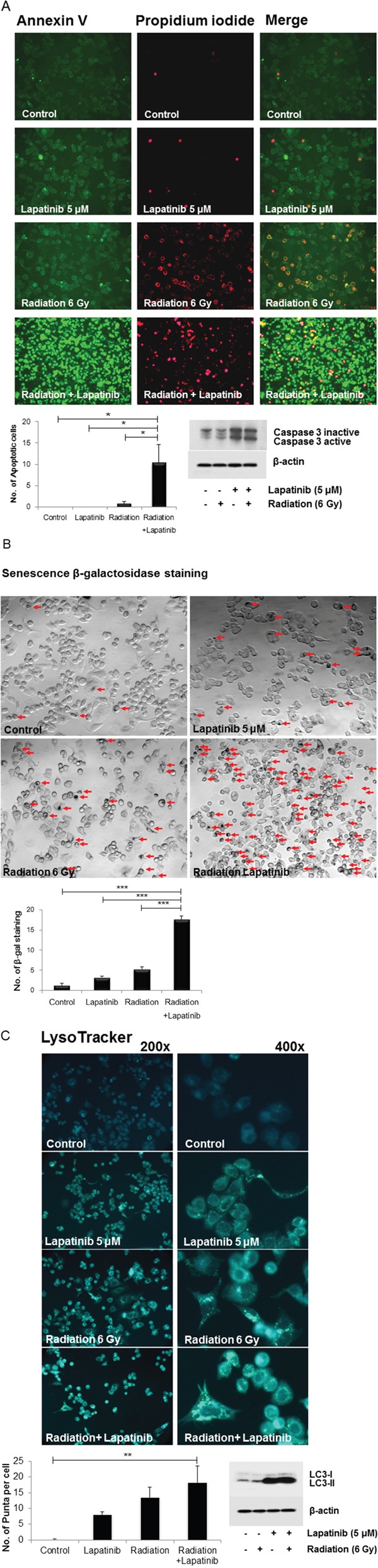
Lapatinib potentiated radiation-induced apoptosis and senescence **A.** SKBR3 cells were treated with lapatinib (5 μM), radiation (6 Gy), or both lapatinib (5 μM) and radiation (6 Gy), after which they were stained with propidium iodide (PI) and antibodies against annexin V. The number of apoptotic cells was determined by foci that were double-stained with annexin V and PI. The number of apoptotic cells was the highest in cells treated with both lapatinib and radiation. Whole-cell extracts were western blotted with the indicated antibodies, and the expression of caspase 3 was increased in cells treated with both lapatinib and radiation. **B.** Senescence was examined by detecting β-galactosidase activity, and a notable change was observed in cells treated with both radiation and lapatinib. **C.** SKBR3 cells were treated with lapatinib (5 μM), radiation (6 Gy), or both lapatinib (5 μM) and radiation (6 Gy), after which they were stained with LysoTracker® Green. The number of Punta formation of radiation+lapatinib-treated cells was higher than that of the other groups. Whole-cell extracts were western blotted with the indicated antibodies, and there was more LC3-I to II conversion when lapatinib was used. ^*^P≤0.05. ^**^P≤0.01. ^***^P≤0.001.

The number of apoptotic cells was determined by annexin V/PI-stained foci. Laptinib combined with radiation significantly increased apoptotic cells and increased expression of cleaved caspase 3 (Figure 3A and supplementary figure S1). Furthermore, combined treatment of lapatinib and radiation showed a notable change in senescence, which was assessed as β-galactosidase activity (Figure 3B). These findings suggest that combined treatment of lapatinib and radiation significantly increased apoptosis and senescence in SKBR3 cells.

To analyze autophagy, cells were stained with LysoTracker^®^ Green. The number of lysosomal localization of cells treated with lapatinib and radiation was higher than that of the other groups, although the difference between cells treated with either lapatinib or radiation and those treated with both lapatinib and radiation was not statistically significant. Lapatinib increased LC-3 I to II conversion (Figure 3C and supplementary figure S2). Either radiation or lapatinib alone showed increased lysosomal localization. Combined treatment of lapatinib and radiation increased autophagy, although the synergistic effect on autophagy was not enough to find statistical significance.

### Lapatinib did not affect the radiosensitivity of HER-2 negative breast cancer cells and normal human astrocytes

**Figure 4:**
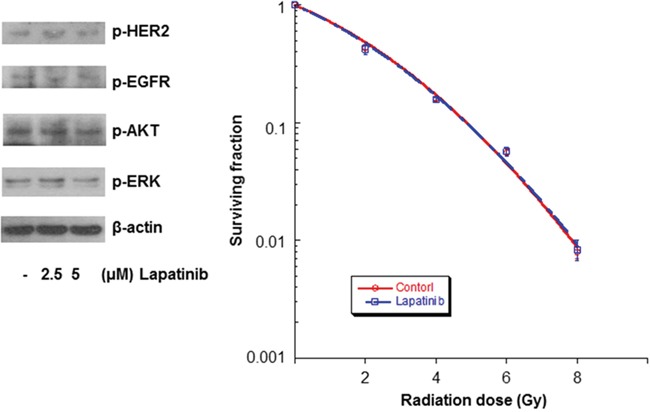
Lapatinib did not affect the radiosensitivity of MCF-7 cells MCF-7 cells were treated with lapatinib, and the cell extracts were western blotted with the indicated antibodies. Lapatinib didn’t affect the expression of p-HER2, p-EGFR, p-AKT, p-ERK in MCF-7 cells. MCF-7 cells were also treated with either DMSO or lapatinib and irradiated with 4-MV x-rays. Lapatinib did not appear to affect the survival fraction of MCF-7 cells.

**Figure 5:**
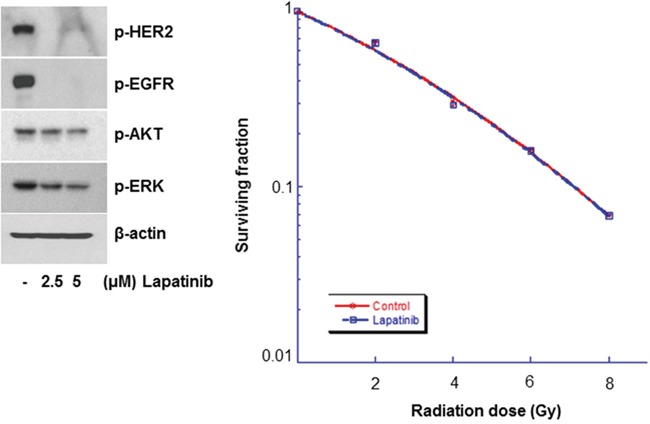
Lapatinib did not affect the radiosensitivity of normal human astrocytes (NHAs) NHAs were treated with lapatinib, and the cell extracts were western blotted with the indicated antibodies. Lapatinib attenuated the expression of p-HER2 and p-EGFR in NHAs. NHAs were also treated with either DMSO or lapatinib and irradiated with 4-MV x-rays. Lapatinib did not appear to affect the survival fraction of NHAs.

To examine the effect of lapatinib on HER-2 negative breast cancer cells, MCF-7 cells were treated with DMSO or lapatinib. Lapatinib did not increase the radiosensitivity of MCF-7 cells and the SERs of the isoeffective dose at surviving fractions of 0.5 and 0.05 were 1.0, respectively (Figure 4). To examine the effect of lapatinib on normal human astrocytes (NHAs) were treated with DMSO or lapatinib. Lapatinib did not increase the radiosensitivity of NHAs and the SERs of the isoeffective dose at surviving fractions of 0.5 and 0.05 were 1.0 (Figure 5).

## DISCUSSION

Overexpression of the HER2 gene results in the formation of a ligand-independent HER2 homodimer and initiate downstream signaling cascades, such as the phosphoinositide 3-kinase (PI3K) and mitogen-activated protein kinase (MAPK) pathways that regulate cell proliferation and survival [22, 23].

Although the anti-proliferative and pro-apoptotic effects of lapatinib have been reported [24, 25], the exact mechanism of radiosensitization and modes of cell death of lapatinib combined with radiation on HER2-overexpressing cells have not been thoroughly elucidated. In this study, we investigated the radiosensitizing effect of lapatinib in HER2+, HER2- breast cancer cells and normal human astrocytes. The mechanism of radiosensitization and modes of cell death were investigated as well. Lapatinib led to downregulation of p-HER2 and p-EGFR, p-Akt and p-ERK, and increased radiosensitivity of breast cancer cells having overexpression of HER2.

Sambade, et al. performed an *in vitro* study using SUM102 cells which overexpress HER2 with normal levels of EGFR. They showed that inhibition of EGFR/HER2 by lapatinib inhibited radiation-induced activation of p-ERK and suggested radiosensitization by lapatinib is mainly mediated by inhibition of MAPK pathway [26]. We speculated that PI3K pathway could also be an important downstream effector contributing radiosensitizing effect of lapatinib as lapatinib down-regulated p-AKT based on the result of our previous study demonstrating that selective inhibition of the PI3K-AKT-mechanistic target of rapamycin (mTOR) pathway radiosensitized HER2-overexpressing cells [27].

Ataxia telangiectasia mutated (ATM) and ATM- and RAD3-related (ATR), which belong to the PI3K-related kinases, respond directly to DNA double-strand breaks (DSBs), enforce cell cycle checkpoints, and participate in the DNA damage response (DDR) [28]. Our study showed that lapatinib inhibited the repair process of DNA DSB as evidenced by the persistence of γH2AX foci and downregulation of p-DNA-PKcs, which is a key enzyme in the NHEJ pathway. Radiation is known to induces multiple pro-survival signaling pathways mediated by AKT and ERK, [29]. Combination of lapatinib can effectively block these pro-survival signaling activated by radiation since lapatinib inhibits both AKT and ERK downstream effector pathways as well.

Apoptosis is the key mechanism by which radiation causes cell death. Lapatinib was able to induce apoptosis of HER2-overexpressing breast cancer cells [30]. Senescence, which is an irreversible arrest of cell proliferation, can also be induced by irradiation. Senescent cells are reproductively inhibited but can be metabolically active and secrete factors called senescence secretome [31]. Angelini et al. showed that the senescence secretomes induced by HER2 carboxyl-terminal fragments are different from those induced by radiation. These secretomes are not inhibited by radiation, but are significantly decreased by the additional lapatinib [31]. We observed that combination of lapatinib significantly enhanced radiation induced apoptosis and senescence.

An autophagy inhibitor, 3-Methyladenine, attenuated autophagy induced by lapatinib in a study using BT474 cells [32]. Other studies have shown that autophagy was an important mode of cell death following lapatinib treatment [33, 34]. Our study also confirmed that either radiation or lapatinib induced autophagy although the synergistic effect was not enough to reach statistical significance.

Radiosensitizing effect of lapatinib was also demonstrated in an *in vivo* study using HER2-positive SUM225 breast cancer cells [35]. After the combination of lapatinib and RT, mouse tumor volumes were significantly reduced in an average SER of 1.25. The radiosensitizing effect of lapatinib was also associated with AKT inhibition in the HER2+ SUM225 model. From a clinical point of view, lapatinib can be used for brain metastases from HER2-positive breast cancer, due to its ability to cross the BBB [12, 36]. When EGFR-overexpressing MDA-MB-231-BR brain-seeking breast cancer cells were injected into a mouse model, lapatinib reduced metastatic colonization in mouse to 50–53% [13]. Because previous studies showed no cross-resistance between trastuzumab-resistant cells and lapatinib-treated cells [30], lapatinib could be used for trastuzumab-resistant patients.

Despite a preclinical study showing the effect of lapatinib on HER2+ cells, lapatinib alone for brain metastasis in HER2-positive breast cancer patients demonstrated limited potential [14–20]. In a phase II trial for patients with HER2-positive breast cancer with brain metastasis, an objective response (defined as ≥50% volume reduction in brain metastases) was achieved in only 6% of the patients [15]. Combination of lapatinib with capecitabine showed higher response rate of 21-66% [16, 18]. Phase I study for WBRT combined with lapatinib involving 35 patients with brain metastasis from HER2-positive breast cancer was reported [37]. Among 28 patients who had measurable brain lesions at baseline, the response rate was 79% (CR in 3 patients and PR in 19 patients).

Based on these findings supporting the radiosensitizing effect of lapatinib, RTOG1119 study which is a phase II randomized study for lapatinib combined with radiation therapy in patients with brain metastases from HER2-positive breast cancer is ongoing (NCT 01622868). Patients are randomly assigned to receive whole brain radiation therapy with or without lapatinib (1000 mg qd). The results of this study would validate the clinical efficacy of lapatinib combined with radiation in patients with brain metastases from HER2-postivie breast cancer.

## MATERIALS AND METHODS

### Cell lines and cell culture

Two HER2-amplified breast cancer cell lines (SKBR3 and BT474, American Type Culture Collection, Rockville, MD, USA) and a HER2-negative breast cancer cell line (MCF-7, American Type Culture Collection, Rockville, MD, USA) were used [38]. Cells were grown in 75-cm^2^ plastic tissue culture flasks at 37°C in Roswell Park Memorial Institute medium (RPMI, Mediatech, Manassas, VA, USA) containing 20% fetal bovine serum (FBS; Invitrogen, Carlsbad, CA, USA) in 5% CO_2_. Immortalized normal human astrocytes (NHAs) were derived from fetal brains.

### Pharmacological inhibitor

Lapatinib ditosylate (GW572016/Tykerb^®^; GlaxoSmithKline, Research Triangle Park, NC, USA) was dissolved into concentrated stock solutions in dimethyl sulfoxide (DMSO), stored at -20°C, and diluted in culture medium. In experimental group, cells were treated with lapatinib for 24 hours. Control cells were treated with medium containing an equal volume of DMSO.

In a previous study, lapatinib (100 mg/kg) was administered to mice via oral gavage. After 2 hours, the blood lapatinib concentration was 5.59±0.56 μM, and the concentration of lapatinib in metastatic brain tissues was 0.71±0.61 μM [21]. In another study, patients with brain metastases from breast cancer received 1,250 mg of oral lapatinib daily for 2–5 days. At the time of brain tumor resection, the serum lapatinib concentration was 2.4–6.5 μM, and the concentration of lapatinib in metastatic brain tissues was 1–63.6 μM [39]. Because of the variability and unpredictability of the BBB in brain metastasis, tissue concentrations are generally not as reliable. Therefore, we chose 5 μM as an optimal concentration of lapatinib for current study which reflected the clinically relevant dose.

### Clonogenic assays

Equal numbers of cells were plated across different treatment groups for each radiation dose. A specified number of cells was seeded into each well of 6-well culture plates and treated with lapatinib 2 hours before irradiation. Lapatinib was exposed for 24 hours. The number of cells seeded was 500 cells at 0 Gy, 1000 cells at 2 Gy, 2000 cells at 4 Gy, 4000 cells at 6 Gy, and 8000 cells at 8 Gy, respectively. The cells were irradiated with 6MV x-rays from a linear accelerator (Clinac 6/100, Varian Medical Systems, Palo Alto, CA, USA) and were incubated for 14–21 days for colony formation. Colonies were fixed with methanol and stained with 0.5% crystal violet. The number of colonies containing at least 50 cells was determined, and the surviving fraction was calculated. Radiation survival data were fitted to a linear-quadratic model using Kaleidagraph version 3.51 (Synergy Software, Reading, PA, USA). Each point on the survival curves represents the mean surviving fraction from at least three dishes. We set the same standard point for both control and lapatinib group as 1 in order to see the radiosensitizing effect effectively. The sensitizer enhancement ratio (SER) was calculated as the ratio of the isoeffective dose at a surviving fraction of 0.5 in the absence of lapatinib to that in the presence of lapatinib. The detailed information is described in our previous report [40].

### Western blot analysis

Cells were washed, scraped, and resuspended in lysis buffer (iNtRON Biotechnology, Seoul, Korea). Proteins were solubilized by sonication, and equal amounts of protein were separated on SDS-PAGE and electroblotted onto polyvinylidene difluoride membranes (Millipore Corp., Bedford, MA, USA). Membranes were blocked in phosphate-buffered saline (PBS) containing 0.1% Tween 20 and 5% powdered milk, after which they were probed with a primary antibody directed against p-HER2 (Tyr1221/1222), p-EGFR (Tyr1068), p-AKT (Ser473), p-extracellular signal-regulated kinase (ERK) (Tyr202/204), p-DNA-PKs (Thr2609), Rad51, caspase3, or LC3 (Cell Signaling Technology, Inc., MA, USA). The monoclonal anti-β-actin antibody was used at a dilution of 1:5,000 (Santa Cruz Biotechnology, Santa Cruz, CA, USA). Membranes were washed and incubated with a secondary antibody consisting of peroxidase-conjugated goat anti-rabbit or anti-mouse immunoglobulin G (Jackson ImmunoResearch Laboratories, West Grove, PA, USA) at a dilution of 1:2,000 for 1 hour. Antibody binding was detected using an enhanced chemiluminescence detection kit (Amersham Biosciences, Piscataway, NJ, USA).

### Immunocytochemistry

Cells were grown and treated on chamber slides. We previously performed the experiments with different time points to investigate DNA damage with γH2AX staining and found that the γH2AX foci formation was most remarkable 24 hours after radiation [41]. Accordingly, cells were fixed 24 hours after the treatment with lapatinib and/or radiation. Cover slips were rinsed, and cells were fixed in 4% paraformaldehyde and permeabilized in methanol for 20 minutes. Cells were subsequently washed and blocked in PBS containing 2% bovine serum albumin for 1 hour. A primary antibody against γH2AX (Cell Signaling Technology, Inc., MA, USA) was applied to the cells and incubated overnight. Secondary fluorescein isothiocyanate anti-rabbit antibody (Molecular Probes, Eugene, OR, USA) was applied and incubated for 2 hours. A 4′,6-Diamidino-2-phenylindole nuclear counter stain was applied at 1 μg/mL for 5 minutes.

For the analysis of autophagy, cells were washed and stained with 1 mM LysoTracker^®^ Green (Molecular Probes, Eugene, OR, USA), which was diluted in PBS, for 10 minutes. Apoptosis was evaluated using annexin V-FITC/propidium iodide (PI) double staining. Cells were stained 24 hours after treatment with lapatinib and/or irradiation. The number of apoptotic cells was determined by annexin V/PI-stained foci. Slides were examined on an Axio Scope.A1 Imager fluorescent microscope. Images were captured and acquired using AxioCam MRc5 and the acquisition software, AxioVision v.4.4 (Carl Zeiss, Gottingen, Germany). The number of formed γH2AX foci and apoptotic cells was counted under 100× magnification, and the number of Punta per cell was counted under 400× magnification.

### β-galactosidase staining

Cellular senescence was evaluated by detecting β-galactosidase activity. Tumor cells were seeded in 8-well chamber slides, treated with lapatinib and/or irradiation, and then stained using a Senescence β-Galactosidase Staining Kit (Cell Signaling Technology, Inc.), according to the manufacturer's instructions. Cells were examined using a light microscope. The number of β-galactosidase-stained cells was counted under 100× magnification.

### Statistical analysis

Data were analyzed for descriptive statistics using SPSS software (SPSS Inc., Chicago, IL, USA). For each variable, the independent t-test was used to evaluate differences. A p value<0.05 indicated statistical significance.

## SUPPLEMENTARY FIGURES AND TABLES


