# Supplementary material for: Radiosensitizing effect of lapatinib in human epidermal growth factor receptor 2-positive breast cancer cells
Source: Oncotarget. 2016 Oct 12;7(48):79089–100. doi: 10.18632/oncotarget.12597 (PMC5346700; doi:10.18632/oncotarget.12597)
Supplement: Supplementary file 1 [file oncotarget-07-79089-s001.pdf]

Radiosensitizing effect of lapatinib in human epidermal growth factor receptor 2-positive breast cancer cells

SUPPLEMENTARY FIGURES

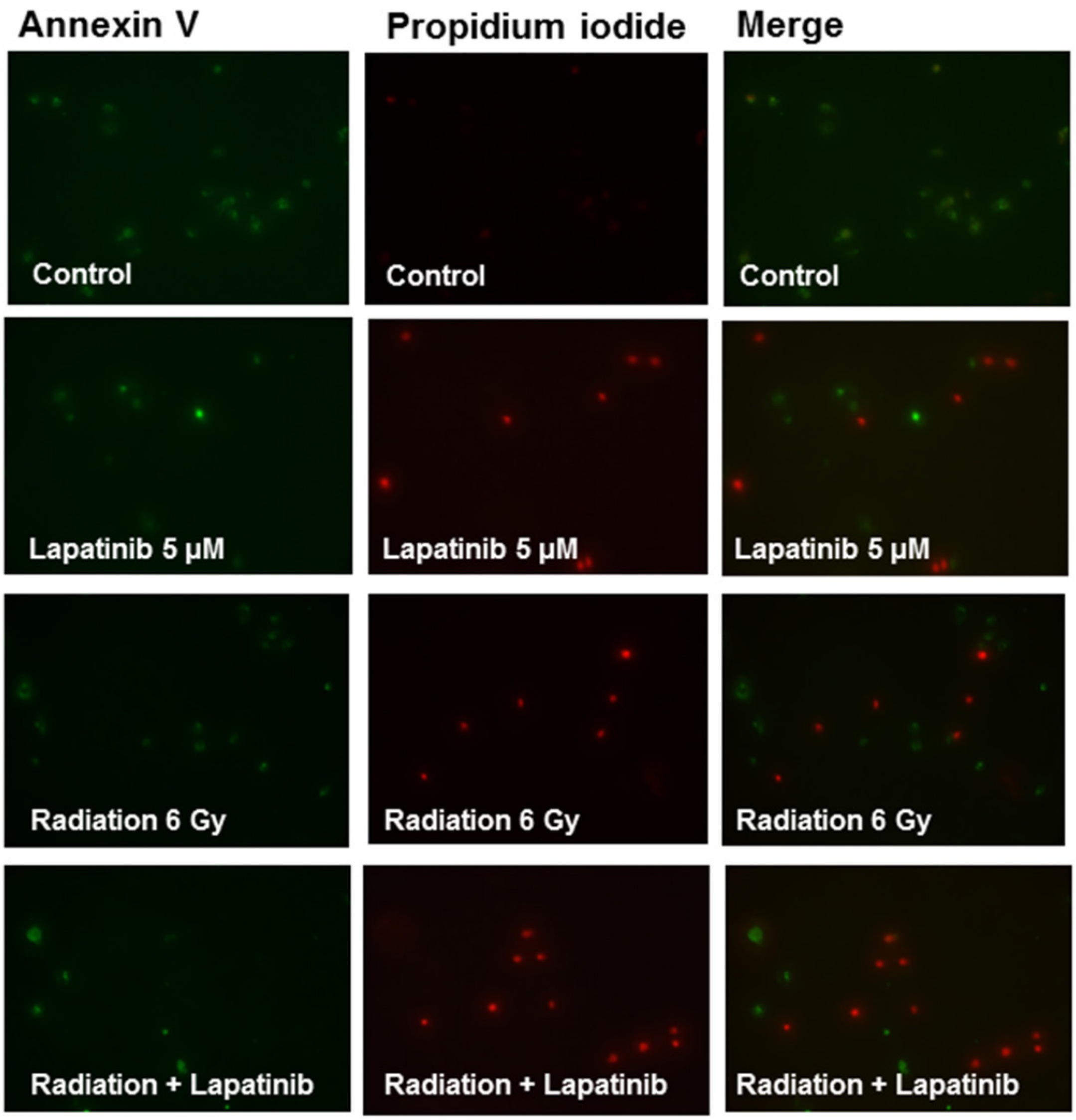

Supplementary Figure S1: SKBR3 cells were treated with lapatinib (5 μM), radiation (6 Gy), or both lapatinib (5 μM) and radiation (6 Gy), and they were stained with propidium iodide (PI) and antibodies against annexin V, 24 hours after the treatment. The number of apoptotic cells was determined by foci that were double-stained with annexin V and PI. The number of apoptotic cells was the highest in cells treated with both lapatinib and radiation.

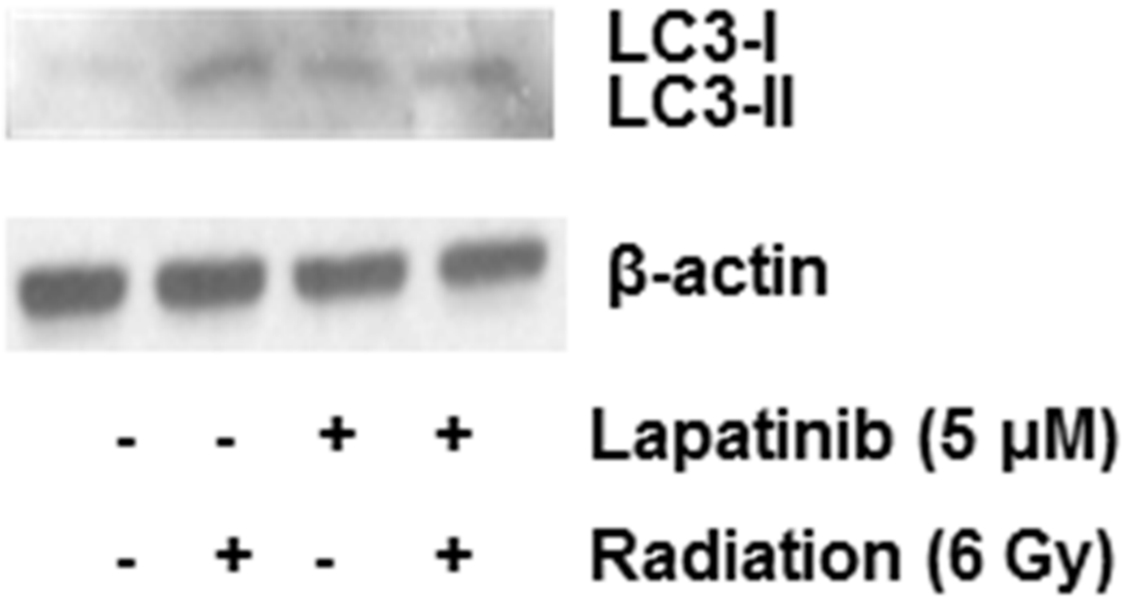

Supplementary Figure S2: SKBR3 cells were treated with lapatinib (5 µM), radiation (6 Gy), or both lapatinib (5 µM) and radiation (6 Gy). Whole-cell extracts were western blotted with the indicated antibodies, 48 hours after the treatment. There was more LC3-I to II conversion when radiation and/or lapatinib were used.
